# Editorial: Residual cardiovascular risk in diabetes: current state and future perspectives

**DOI:** 10.3389/fcdhc.2026.1774240

**Published:** 2026-02-18

**Authors:** Joaquim Oliveira, Thiago Qunaglia, Andrei C. Sposito

**Affiliations:** Laboratory of Atherosclerosis and Vascular Biology, Faculty of Medical Sciences, UNICAMP, Campinas, Brazil

**Keywords:** cardiovascular prevention, cardiovascular risk, diabetes, lifestyle and behavior, SGLT2

Patients with type 2 diabetes experience threefold higher risk of cardiovascular disease and mortality compared with the general population (1). Goal-oriented management of hypertension, glucose levels and dyslipidemia reduces but does not eliminate excess cardiovascular risk, with individuals with strictly controlled comorbidities displaying 21% higher incidence of cardiovascular events compared with healthy individuals (2). This persistent vulnerability, namely residual risk, results from a constellation of thrombotic, inflammatory, and metabolic pathways lying outside the reach of conventional therapies (2). With current estimates projecting nearly 4 million annual deaths attributable to diabetes, identifying novel biomarkers for broader risk stratification and the pursuit of agents with pleiotropic properties capable of addressing pathways untouched by conventional risk-factor control have emerged as a critical priority (3). This research topic includes clinical evidence and mechanistic insights to advance understanding and clarify translational opportunities relevant to cardiometabolic care.

Sodium-glucose cotransporter 2 inhibitors (SGLT2i) have reshaped the landscape of diabetes care in recent years (4). Though initially designed for glucose-lowering, robust randomized trials have demonstrated one-third consistent risk reductions in major cardiovascular events including cardiovascular death, all-cause mortality and hospitalization for heart failure (4). Gliflozins have also prevented kidney function decline and the progression of proteinuria in chronic kidney disease populations, thereby mitigating the burden of diabetic kidney disease and kidney failure (4). With population-level projections of 5-year rescue of life expectancy, the clinical benefit of this class beyond glycemic control includes weight loss and improvements in myocardial function and fluid balance (5). As a valuable addition to this remarkable cardiorenal benefit, gliflozins promote sustained blood pressure reduction that, though modest compared with antihypertensive agents, has been recognized as a potential adjunct therapy for management of hypertension (6). Blood pressure lowering in this context emerges from intricate mechanisms encompassing neurohormonal effects, insulin resistance, osmotic diuresis, and gene polymorphisms (7). These mechanisms and the role of gliflozins in reducing residual risk through blood pressure lowering are reviewed in this issue.

Timely identification of high-risk patients using standardized cardiovascular risk equations based on traditional predictors has been a cornerstone strategy of preventive cardiology (8). Incorporating novel residual risk biomarkers permits discriminatory power and reclassification capacity refinements through broader assessment of systemic vulnerability to cardiovascular disease (8, 9). Glycated hemoglobin has been the most utilized metabolic stress biomarker in type 2 diabetes showing a graded association with diabetes-related complications (10). This metric has nonetheless limited capacity to capture glycemic variability, which has incremental prognostic value compared to glycated hemoglobin by reflecting patients physiologic response to acute metabolic stress beyond chronic glycemic control (11). In this issue, Lian et al. contribute to this evolving concept by demonstrating an independent association of stress hyperglycemia ratio, hereby calculated diving post-cardiac arrest glycemia by glycated hemoglobin, with 1-year mortality rates using data from 535 intensive care unit patients enrolled in the MIMIC-IV database. This research topic also presents the results of Kong et al. retrospective single-cohort of 201 adults with type 2 diabetes who completed flow-mediated dilation (FMD) and serum soluble TREM-1 (sTREM-1) levels assessments. sTREM-1, a myeloid innate immunity receptor linked to low-grade lasting vascular inflammation, was independently associated with lower FMD, thereby suggesting the interplay between this reliable marker of residual inflammatory risk and endothelial dysfunction.

Diabetes care guidelines endorse structured interdisciplinary lifestyle programs for broad metabolic risk lowering beyond goal-directed pharmacotherapy (8). This recommendation is grounded by randomized clinical trial (eg., Look-AHEAD study) evidence of sustained improvements in glycemic control, lipid profile, body weight, and overall quality of life and fitness with healthier diets, diabetes education, and physical activity (12, 13). Emerging evidence supports that replacing saturated fats with dietary polyunsaturated fats (PUFA) could improve residual cardiometabolic risk attributable to nutritional factors with established effects on cholesterol levels, gut microbiota, and liver steatosis (14, 15). Consistently, Jiang et al. hereby demonstrated an inverse association between PUFA dietary intake (linoleic acid) and cardiovascular mortality among 3,112 adults with type 2 diabetes enrolled in the NHANES cohort, whereas Høgsholt et al. single-center retrospective cohort have found lower body weight and glucose-levels among patients with type 2 diabetes who have completed a 6-month supervised lifestyle interventions.

This research topic therefore gathers compelling evidence of the critical importance of residual cardiovascular risk identification and management to improve clinical outcomes in type 2 diabetes. Expanding clinical use of emerging biomarkers of systemic vulnerability —such as stress hyperglycemia and sTREM-1— could assist earlier detection of high-risk individuals who could benefit the most from stricter clinical control. Prescription of antidiabetic agents with pleiotropic properties, in addition to structured lifestyle modifications including appropriate dietary guidance plays a critical role in addressing the emerging global challenge of type 2 diabetes cardiovascular morbidity. These concepts and mechanistic insights are hereby developed.
